# Systematic variations associated with renal disease uncovered by parallel metabolomics of urine and serum

**DOI:** 10.1186/1752-0509-6-S1-S14

**Published:** 2012-07-16

**Authors:** Xianfu Gao, Wanjia Chen, Rongxia Li, Minfeng Wang, Chunlei Chen, Rong Zeng, Yueyi Deng

**Affiliations:** 1 Key Laboratory of Systems Biology, Shanghai Institutes for Biological Sciences, Chinese Academy of Sciences, 320 Yue Yang Road, Shanghai 200031, China; 2 Department of Nephrology, Longhua Hospital, Shanghai University of Traditional Chinese Medicine, 725 Wanping Road, Shanghai, 200032, China

## Abstract

**Background:**

Membranous nephropathy is an important glomerular disease characterized by podocyte injury and proteinuria, but no metabolomics research was reported as yet. Here, we performed a parallel metabolomics study, based on human urine and serum, to comprehensively profile systematic metabolic variations, identify differential metabolites, and understand the pathogenic mechanism of membranous nephropathy.

**Results:**

There were obvious metabolic distinctions between the membranous nephropathy patients with urine protein lower than 3.5 g/24 h (LUPM) and those higher than 3.5 g/24 h (HUPM) by Partial Least Squares Discriminant Analysis (PLS-DA) model analysis. In total, 26 urine metabolites and 9 serum metabolites were identified to account for such differences, and the majority of metabolites were significantly increased in HUPM patients for both urines and serums. Combining the results of urine with serum, all differential metabolites were classified to 5 classes. This classification helps globally probe the systematic metabolic alterations before and after blood flowing through kidney. Citric acid and 4 amino acids were markedly increased only in the serum samples of HUPM patients, implying more impaired filtration function of kidneys of HUPM patients than LUPM patients. The dicarboxylic acids, phenolic acids, and cholesterol were significantly elevated only in urines of HUPM patients, suggesting more severe oxidative attacks than LUPM patients.

**Conclusions:**

Parallel metabolomics of urine and serum revealed the systematic metabolic variations associated with LUPM and HUPM patients, where HUPM patients suffered more severe injury of kidney function and oxidative stresses than LUPM patients. This research exhibited a promising application of parallel metabolomics in renal diseases.

## Background

Membranous nephropathy (MN) is an important glomerular disease characterized by podocyte injury and proteinuria. Although the definite pathogenic mechanisms of MN has not yet been entirely clarified, it is widely accepted that MN involves the in situ formation of subepithelial immune complex deposits and subsequent complement activation leading to podocyte injury and diffuse thickening of the glomerular basement membrane, based on experimental animal models and human studies [1-3]. The diagnosis and treatment of MN patients are presently based mainly on clinical manifestations, urinary protein excretion levels, and renal biopsy. Renal biopsy, though providing specific diagnosis currently, is an invasive procedure with considerably significant complications, particularly in patients with bleeding tendency or skin infection on the flank. Thus, a renal biopsy may be contraindicated for certain high risk patients, and was often refused by early patients. At the stage of MN, the function of glomerular filtration can be kept in a normal range. Routine clinical chemical measures of renal function based on blood, such as serum creatinine, serum uric acid, albumin, and total protein, are insensitive and laggard for early diagnosis. They elevated significantly only after substantial kidney injury occurs, generally after a loss of two third or greater of nephron functional capacity [4]. However, the metabolites that are overproduced during MN progression will enter urine space and be secreted into final urine. For this reason, urine possibly is an ideal source of test materials to noninvasively characterize the activity of kidney.

Metabolomics, an important constituent of systems biology, aims to simultaneously measure as many metabolites as possible in a given biological system in order to acquire an overview of metabolic status and global biochemical events associated with a cellular or biological system [5]. It is well known that the minor alteration at the level of gene or protein expression usually leads to significant change in metabolite level. Combining a robust instrumental analysis with whole metabolite information and multivariate statistical analysis, such as principal component analysis (PCA) and partial least squares discriminant analysis (PLS-DA), metabolomics has been a considerably intensive means for comprehensively evaluating toxicity of drugs or xenobiotics [6,7], early diagnosis and identifying potential biomarkers [8,9], and elucidating biological pathways [10]. Mass spectrometry (MS) and nuclear magnetic resonance (NMR) spectroscopy are major analytical tools for metabolomics. MS coupled with advanced chromatographic separation instruments, such as gas chromatography (GC) or liquid chromatography (LC), has become a powerful metabolomics tool, with a wide dynamic range and reproducible quantitative performance, for the measurement of complex biological samples. GC/MS is a robust analytical platform for quantification with better sensitivity and resolution than the commonly used NMR approach and better reliability in structure identification of candidate biomarkers than LC/MS [11]. It has been widely applied in metabolomics research of biofluids, feces, and tissue samples [6,12-14]. Nowadays, metabolomics has been successfully used in the fields of physiology, diagnostics, functional genomics, pharmacology, toxicology, and nutrition. Furthermore, simple and non-invasive sample collection techniques, fast instrumental analysis, and meaningful diagnostic information enable urine to be a considerably suitable biofluid for metabolomics analysis in personalized medicine such as the recent application in personalized neonatal medicine [15].

Urine-based metabolomics has been applied in the researches of early detection and renal toxicity [6,9], diagnosis, biomarker identification, and pathogenic pathway of kidney cancer [8,16]. To our knowledge, there is no research work published about MN based on metabolomics. In omics field, there is only a paper reported the changes of urinary proteome profile using a MN animal model [17]. In the present study, we adopted a GC/MS-based metabolomics approach, based on clinical urine and serum samples, to systematically evaluate the metabolic alterations and understand the differential pathological characteristics between the MN patients with urine protein lower than 3.5 g/24 h and those with urine protein higher than 3.5 g/24 h. Ongoing work in our lab is directed to further study of such disease at a larger sample scale and validate the potential biomarkers for the researches of clinical diagnosis and pathological mechanism.

## Results

### Histopathology and clinical chemistry

The diagnosis and grading of MN patients were performed according to clinical chemical parameters and renal biopsy. The patients were clinically classified to *L*ow *U*rine *P*rotein *M*N group (LUPM, urine protein < 3.5 g/24 h) and *H*igh *U*rine *P*rotein *M*N group (HUPM, urine protein > 3.5 g/24 h). According to the detailed PCA analysis in the next section, 3 samples with serum creatinine higher than 110 μmol/l but urine protein lower than 3.5 g/24 h were found to be outliers, and were excluded from final statistics of clinically chemical parameters. Table 1 lists the clinical parameters. Although the average urine creatinine level of HUPM group was higher than that of LUPM group, there was no statistical difference between them. It was worth noting that the serum creatinine of HUPM group was significantly higher than that of LUPM group, suggesting the different glomerular filtrate rate and renal function between the two groups of MN patients. Obviously, the serum creatinine clearance by kidneys of HUPM patients was weaker than LUPM patients, but the creatinine values of both two groups were still within the normal range. In general, the glomerular basement membrane with normal function is negatively charged in the body, which prevents the filtration of the negatively charged proteins such as albumin to the urine. In contrast to the LUPM group, the markedly reduced serum albumin and total protein in HUPM group possibly reflected the increased damage of glomerular basement membrane.

**Table 1:** Clinically chemical analysis of membranous nephropathy

Parameters	LUPM (*n *= 14)	HUPM (*n *= 15)	*p*-value *
Urine output (ml/24 h)	2370.8 ± 840.4	2408.5 ± 401.2	0.885
Urine protein (g/24 h)	1.42 ± 1.07	5.17 ± 1.70	< 0.001
Urine creatinine (mmol/24 h)	8.91 ± 6.62	11.59 ± 3.08	0.211
Serum creatinine (Scr, μmol/l)	58.89 ± 15.0	85.4 ± 31.8	0.039
Serum total protein (g/l)	60.2 ± 12.6	45.9 ± 12.6	0.023
Serum albumin (g/l)	34.0 ± 8.7	22.6 ± 7.5	0.006
Blood urea nitrogen (BUN, mmol/l)	4.7 ± 1.8	6.4 ± 2.5	0.115

### Urinary metabolomics analysis

**Figure 1:**
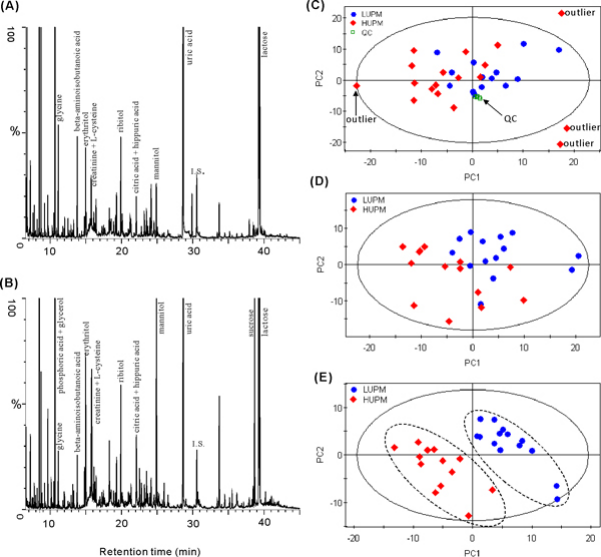
**Representative urinary GC/MS total ion current (TIC) chromatograms and the scores plots of PCA and PLS-DA**. (A), the representative GC/MS TIC chromatogram of LUPM patient urine; (B), the representative GC/MS TIC chromatogram of HUPM patient urine; (C), PCA scores plot of QC (square), LUPM (dot), and HUPM (diamond) urines with outliers, QC sample was prepared by pooling the urine samples in this study; (D), PCA scores plots of LUPM (dot) and HUPM (diamond) urines after excluding outliers; (E), PLS-DA scores plots of LUPM (dot) and HUPM (diamond) urines after excluding outliers.

Trimethylsilyl derivatization was applied in sample preparation prior to GC/MS analysis. This derivatization can efficiently improve GC/MS analysis of the compounds which contain carboxylic, amino, or hydroxyl group, such as fatty acids, amino acids, amines, hydroxyl acids, carbohydrates, and phenolic acids. The representative urinary GC/MS total ion current (TIC) chromatograms of the LUPM and the HUPM patients were displayed in Figure 1A and 1B, respectively. A total of 512 chromatographic peaks were resolved from over 80% of samples after the exclusion of known internal standard and artifact peaks. In order to obtain more reliable outcomes at the level of population, multivariate statistical analysis, such as PCA and PLS-DA, was performed on the preprocessed GC/MS data matrices. PCA was initially performed on the normalized peak areas obtained from all the samples to evaluate the quality of sample analysis and view the holistic distribution, clustering, and outlier of samples. The PCA scores plot (2 principal components, R^2^X = 0.269, Q^2 ^= 0.109, Figure 1C) shows that all QC samples, prepared by pooling the urine samples in this study, are tightly clustered in a small area, demonstrating that current protocol is reliable and thereby the variance derived from metabolomics analysis can be ignored at the following data analysis [18]. Four samples were out of the Hotelling T^2 ^95% confidence, and thus were suspected as outlier samples. After checking the clinically chemical parameters, it was found that all of the 3 samples with serum creatinine higher than 110 μmol/l but urine protein lower than 3.5 g/24 h were involved in the outliers. In addition, the outlier samples with high serum creatinine distributed in scattered space of PCA scores plot. The three samples and another one outlier sample were excluded at the subsequent analysis.

After removing 4 outlier samples, PCA model (2 components, R^2^X = 0.229, Q^2 ^= 0.041) was constructed again, and the scores plot shows that no evident separation but a separation tendency indeed exists between the LUPM group and HUPM group (Figure 1D). R^2 ^and Q^2 ^values were calculated for the model to assess any significance of differentiation and the quality of model. The low statistical parameters showed that PCA model was not suitable for interpreting the differences. A PLS-DA model with 3 components was thus conducted to reveal the differences between the two groups. The PLS-DA model showed significantly improved model predictability (Q^2 ^= 0.657), and fairly good capability to explain the metabolic variation between LUPM and HUPM patients (R^2^Y = 0.977). Figure 1E indicates that the two groups are evidently separated in PC1 and PC2. The PLS-DA scores plot implied that the severity of MN patients could be discriminated according to the urine protein level.

The differential metabolites contributing to the metabolic differences between the LUPM group and HUPM group were further identified. Among the statistically significant variables, 26 differential metabolites were structurally qualified using reference compounds and NIST library with retention indexes http://www.sisweb.com/software/ms/nist.htm. These metabolites were summarized in Table 2.

**Table 2:** Differential urinary metabolites between HUPM and LUPM patients

**No**.	Compounds	VIP *^a^*	*p*-Value *^b^*	FDR *^c^*	FC *^d^*	Biochemical pathway
1	cis-Aconitic acid	1.73	9.87E-03	2.42E-02	-0.74	TCA cycle
2	Lactose	1.55	2.23E-02	3.54E-02	-0.45	Galactose metabolism
3	Erythritol	1.72	9.99E-03	2.25E-02	0.41	Polyol metabolism
4	Xylitol	1.39	4.27E-02	4.80E-02	0.30	Pentose and glucuronate interconversions
5	Galactitol	1.38	4.86E-02	4.86E-02	0.62	Galactose metabolism
6	Inositol	1.73	9.63E-03	2.60E-02	1.00	Galactose metabolism
7	Glyceric acid	1.52	2.54E-02	3.43E-02	0.69	Glyoxylate and dicarboxylate metabolism
8	2,4-Dihydroxybutyric acid	1.74	9.20E-03	2.76E-02	0.44	Fatty acid metabolism
9	Threonic acid	2.17	7.40E-04	6.66E-03	0.35	Ascorbate and aldarate metabolism
10	2-Deoxyribonic acid	1.58	1.97E-02	3.32E-02	0.39	
11	2-Ketogluconic acid	1.71	1.07E-02	2.22E-02	0.41	
12	Glutaric acid	1.66	1.37E-02	2.47E-02	0.48	Fatty acid metabolism, Lysine degradation
13	3-Methylglutaric acid	1.74	9.12E-03	3.08E-02	0.48	Leucine metabolism
14	Adipic acid	2.17	6.97E-04	9.41E-03	0.58	Fatty acid metabolism
15	2-Hydroxyglutaric acid	1.94	3.13E-03	1.41E-02	0.40	Fatty acid metabolism
16	Suberic acid	1.70	1.30E-02	2.51E-02	0.54	Fatty acid metabolism
17	3-Hydroxysebacic acid	1.56	2.36E-02	3.54E-02	0.80	Fatty acid metabolism
18	Mandelic acid	1.44	3.47E-02	4.46E-02	0.50	Aminobenzoate degradation
19	4-Hydroxyphenylacetic acid	1.41	3.86E-02	4.53E-02	0.37	Phenylalanine and tyrosine metabolism
20	Vanillic acid	1.36	4.70E-02	4.88E-02	0.25	Gut microflora metabolism
21	3,4-Dihydroxybenzoic acid	1.42	3.83E-02	4.70E-02	0.69	phenylalanine, tyrosine and tryptophan biosynthesis
22	4-Hydroxyphenyllactic acid	1.52	2.53E-02	3.60E-02	0.49	Phenylalanine and tyrosine metabolism
23	Vanillactic acid	2.04	1.66E-03	1.12E-02	1.15	Gut microflora metabolism
24	Cytosine	1.91	3.71E-03	1.43E-02	0.81	Pyrimidine metabolism
25	Quinolinic acid	2.31	2.53E-04	6.83E-03	0.61	Nicotinate and nicotinamide metabolism
26	Cholesterol	1.97	2.70E-03	1.46E-02	1.51	Steroid biosynthesis

*^a ^*Variable importance in the projection (VIP) was obtained from PLS-DA model with value higher than 1.0;

*^b ^*The *p*-value calculated from two-tailed Student's *t *test;

*^c ^*FDR adjusted *p *value from two-tailed Student's *t *test;

*^d ^*Fold change was calculated as binary logarithm of average mass response (normalized peak area) ratio between HUPM and LUPM patients, where the positive value means that the average mass response of the metabolite in the HUPM group is larger than that in the LUPM group.

They mainly included dicarboxylic acids, hydroxyl acids, phenolic acids, TCA cycle intermediates, sugar alcohols, cytosine, quinolinic acid, and cholesterol. In contrast to LUPM subjects, the majority of metabolites were increasingly excreted in the urines of HUPM patients. Dicarboxylic acids are generally the products of polyunsaturated fatty acids under oxidative stress. Sugar alcohols are commonly as the regulator of the osmotic pressure during the formation of urine. The overproduction of this kind of metabolites in the urines of HUPM patients indicated that the re-absorption function and self metabolism of their kidney possibly received more severe injury than LUPM subjects.

### Metabolomics analysis of serum samples

**Figure 2:**
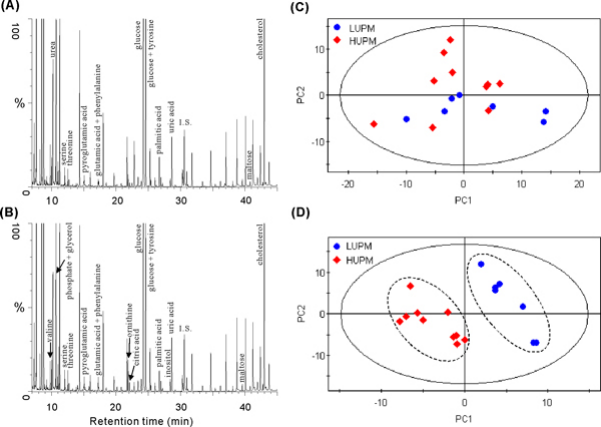
**Representative serum GC/MS TIC chromatograms and the scores plots of PCA and PLS-DA**. (A), the representative serum GC/MS TIC chromatogram of LUPM patient; (B), the representative serum GC/MS TIC chromatogram of HUPM patient; (C), PCA scores plots of LUPM (dot) and HUPM serums (diamond); (D), PLS-DA scores plots of LUPM (dot) and HUPM serums (diamond).

Serum samples were simultaneously analyzed in this study. With the consent of patients, we collected the serum samples of a part of volunteer patients. The representative serum GC/MS total ion current (TIC) chromatograms of the LUPM patient and the HUPM patient were shown in Figure 2A and 2B, respectively. Totally, 217 chromatographic peaks were obtained from the over 80% of samples after exclusion of known internal standard and artifact peaks. Similar to urine metabolomics analysis, we first performed a PCA model to validate the robustness of our metabolomics protocol. After exclusion of QC samples, a new PCA model was performed again. The PCA scores plot (Figure 2C) indicates that, similar to urine, no clear profile separation was present between the two groups of samples (2 components, R^2^X = 0.366, Q^2 ^= 0.081). The small values of statistical parameters indicated that PCA model cannot explain the differences between the serum samples of LUPM and HUPM patients. Subsequently, a PLS-DA model was conducted to reveal variations between the two groups. The parameters (2 components, R^2^Y = 0.935, Q^2 ^= 0.639) indicated that the PLS-DA model was reliable to explain the phenotype difference between the LUPM and HUPM patients. The PLS-DA scores plot demonstrates that the two groups of samples are well separated to two clusters, thus the LUPM group was different from the HUPM group by metabolomics analysis of serum samples (Figure 2D).

The serum metabolites contributing to such metabolomics profile separation were identified. Nine serum metabolites were annotated by the NIST library, and of which 7 metabolites were confirmed by our reference compounds (Table 3). The number of differential metabolites from serum is obviously less than that from urine. Similar to the urine results, cholesterol was found significantly increased in the HUPM group. However, unlike urine, the citric acid level of HUPM group was significantly higher than that of LUPM group. No urinary amino acids were found differentially expressed between the LUPM and the HUPM patients, but 4 serum amino acids were significantly increased in the HUPM patients.

**Table 3:** Differential serum metabolites between HUPM and LUPM patients

**No**.	Compound	VIP *^a^*	*p*-Value *^b^*	FDR *^c^*	FC *^d^*	Biochemistry pathway
1	m-Cresol	1.93	1.45E-02	2.61E-02	-1.66	Gut microflora metabolism
2	2-Keto-3-methylvaleric acid	2.31	4.47E-03	1.34E-02	-0.58	Fatty acid metabolism
3	L-Asparagine ^#^	2.34	1.40E-03	6.30E-03	0.40	Alanine, aspartate and glutamate metabolism
4	L-Serine ^#^	2.46	1.04E-03	9.36E-03	0.79	Glycine, serine and threonine metabolism
5	L-Threonine ^#^	1.90	1.47E-02	2.21E-02	0.56	Glycine, serine and threonine metabolism
6	Pyroglutamic acid ^#^	1.63	3.60E-02	4.05E-02	0.27	Glutathione metabolism
7	Citric acid ^#^	1.96	1.35E-02	3.04E-02	0.85	TCA cycle
8	Glucose ^#^	1.53	4.80E-02	4.80E-02	0.44	Glycolysis, Pentose phosphate pathway, Galactose metabolism
9	Cholesterol ^#^	1.82	2.07E-02	2.66E-02	0.41	Steroid biosynthesis

*^a ^*Variable importance in the projection (VIP) was obtained from PLS-DA model with value higher than 1.0;

*^b ^*The p-value was calculated from Student's *t *test;

*^c ^*FDR adjusted *p *value from two-tailed Student's *t *test;

*^d ^*Fold change was calculated as binary logarithm of average mass response (normalized peak area) ratio between HUPM and LUPM patients, where the positive value means that the average mass response of the metabolite in the HUPM group is larger than that in the LUPM group;

^# ^The metabolite was structurally confirmed by available reference compound.

## Discussion

The metabolites that are massively overproduced during the progression of membranous nephropathy and fit the criteria of glomerular filtration, including blood and the metabolites of kidney, will enter urine space and the final urine. In this study, the routine clinical chemical measures of renal function indicated that the serum creatinine of almost all patients were kept in normal range, suggesting that the function of glomerular filtration probably was not substantially altered, irrespective of glomerular injury happened actually. Here, we performed a parallel metabolomic study based on urine and serum to globally profile metabolic differences and identify differential metabolites, and attempted to understand the pathogenic mechanism of membranous nephropathy based on different urine protein levels.

**Figure 3:**
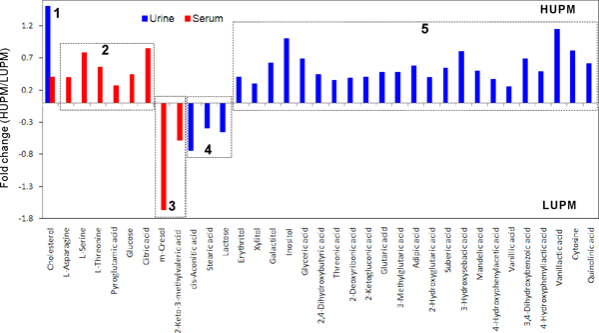
**Trend map of differential metabolites in urine (blue) and serum (red) between LUPM and HUPM patients**. Horizontal axis represents metabolites, and vertical axis represents fold change between HUPM and LUPM (HUPM/LUPM). The bar above zero axis means the concentration of this metabolite is higher in HUPM patients than in LUPM patients. The bar below zero axis means the concentration of this metabolite is higher in LUPM patients than in HUPM patients. If the metabolite was not significantly changed between LUPM and HUPM patients, it was blank. Fold change was calculated as binary logarithm of average mass response (normalized peak area) ratio between HUPM and LUPM patients, where the positive value means that the average mass response of the metabolite in the HUPM group is larger than that in the LUPM group. All of these differential metabolites were classified to 5 groups according to their change trend in urine and serum.

It is important to compare the differential metabolites before kidney (ie. serum) and after kidney (ie. urine). The number of differential urinary metabolites was markedly more than that of differential serum metabolites between the patients of LUPM and HUPM. The levels of the majority of differential metabolites were significantly increased in HUPM patients for both urines and serums. Figure 3 demonstrates that all differential metabolites can be classified to 5 groups according to their change trend in urine and serum. Cholesterol was higher in both urine and serum of HUPM patients (Class 1). Four amino acids were found as differential serum metabolites with markedly increased levels in HUPM patients, while they were not differentially excreted in the urines (Class 2). Class 2 reflects that the kidneys of HUPM patients possibly possessed weaker filtration ability for amino acids and citric acid, and thus suffered more severe injury than those of LUPM patients. It was presumed that these elevated 4 amino acids and citric acid in serum samples of HUPM patients probably came from the metabolism of other organs (via blood) rather than kidney, and were to some extent prevented into urine by impaired kidney. 2-Keto-3-methylvaleric acid and m-cresol were higher in the serums of LUPM patients and classified to Class 3, but their functions remain unclear. The Class 4 contains lactose and cis-aconitic acid with higher secretion only in the urines of LUPM patients. Other differential metabolites increased only in urines of HUPM patients (Class 5), such as sugar alcohols, dicarboxylic acids, hydroxyl acids, and phenolic acids, are possibly derived from the metabolism of kidney, because there were no differences between the serum samples of LUPM and HUPM patients. Thus, the metabolites of Class 5 probably were the direct outcomes of renal injury.

Four sugar alcohols (polyols) were increasingly excreted in the urines of HUPM patients, while their concentrations were not changed significantly in the serum samples. Polyols are polyhydric alcohols, derived from aldoses and ketoses by reduction with NADPH. In this study, the serum and urine glucose levels of HUPM patients were significantly higher than those of LUPM subjects (no statistical significance for urinary glucose, *p *= 0.058), suggesting that these polyols possibly came from the elevated glucose. Recent research proved that erythritol was an excellent antioxidant [19], and attenuated the diabetic oxidative stress [20].

**Figure 4:**
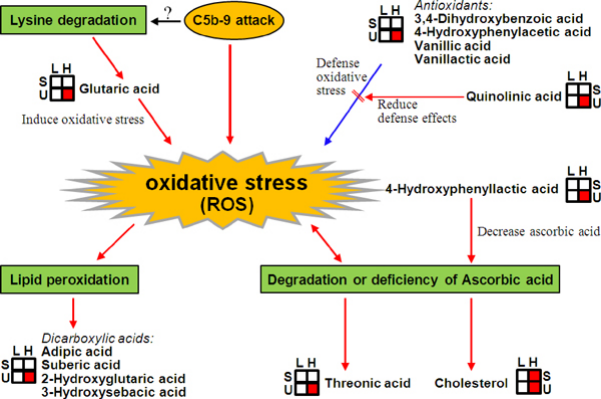
**Proposed metabolic network associating with increased oxidative stress during the developing of membranous nephropathy**. C5b-9 attack, the attack from membranous protein complex C5b-9 on podocyte; ROS, reactive oxygen species; L, Low urine protein membranous nephropathy; H, High urine protein membranous nephropathy; S, serum; U, urine; Red box means that the metabolite in urine or serum of "H" group was increased significantly than "L" group.

The increasing evidence shows that oxidative stress formed in podocyte response to the membrane attack complex C5b-9 induced injury is an important outcome. The attack of C5b-9 on podocytes induces production of reactive oxygen species (ROS), which initiate lipid peroxidation and subsequent degradation of glomerular basement membrane collagen IV, leading to development of proteinuria [21]. In this research, the majority of metabolites in Class 1 and 5 were presumed to be closely related to the increased oxidative stress. According to previous knowledge, we proposed a metabolic network associating with the increased oxidative stress during the developing of membranous nephropathy (Figure 4).

In this study, a series of dicarboxylic acids and corresponding hydroxyl metabolites, such as adipic acid, suberic acid, and 3-hydroxysebacic acid, were excessively excreted in the urines of HUPM patients, compared with LUP patients. Bergseth et al [22] found that urinary dicarboxylic acids might to a significant extent be formed in the kidneys themselves. Glutaric acid, a product of lysine degradation, can induce oxidative stress in brain of young rat [23]. Under pathological status, free or esterified cis-polyunsaturated fatty acids generated saturated short- (ie. adipic acid) and medium-chain length dicarboxylic acids (ie. suberic acid) due to peroxisomal disorder by oxidative attack [24]. The elevated urinary (hydroxyl)dicarboxylic acids in HUPM patients possibly reflected an more severe oxidative stress than LUPM patients in this study.

Threonic acid is another one metabolite of interest. The formation of this metabolite is closely related to the deficiency or degradation of ascorbic acid, a potent water-soluble antioxidant that readily scavenges free radicals such as molecular oxygen, superoxide, and hydroxyl radical [25]. Threonic acid is related to the protein collagen, but its function in nephropathy hitherto was still not reported. The higher level of urinary threonic acid in HUPM patients than LUPM patients indirectly indicated the significantly strengthened oxidative stress in HUPM patients. It is known that ascorbic acid helps decrease cholesterol. The increased urinary and serum cholesterol levels in the HUPM patients further proved the deficiency of ascorbic acid and hence the increased oxidative stress.

Previous proteomics research based on a rat MN model found that aldehyde dehydrogenase, which is capable of converting 9-cis and all-trans retinal to corresponding retinoic acid with high efficiency, was decreased [17]. Its function has been suggested to involve in oxidative pathway [26]. It is known that HUPM patients clinically are more severe than LUPM patients. Metabolomics analysis in this research proved the more strengthened oxidative stress, mainly in kidney, during the pathogenesis and progression of HUPM patients. Currently, studies that used antioxidants and oxygen radical scavengers have demonstrated beneficial effects in animal model and human MN treatments [27,28].

A lot of phenolic acids were increasingly excreted in the urines of HUPM patients. They are the metabolites of phenylalanine and tyrosine metabolism and usually serve as antioxidants [29,30]. In contrast to LUPM patients, the increased 3,4-dihydroxybenzoic acid, 4-hydroxyphenylacetic acid, vanillic acid, and vanillactic acid in the urines of HUPM patients suggested that more antioxidants were possibly produced and transferred into glomerulus to defense the increasing oxidative stress during pathological progression, and thereafter excreted massively in urines. In this study, however, we did not know whether these phenolic acids were primarily formed in kidney or in other organs, because no differences were found between the serum samples of the two groups of patients.

A phenolic acid of special interest is 4-hydroxyphenyllactic acid, which was increasingly excreted in the urines of HUPM patients. It is a carcinogenic metabolite of the disorder of tyrosine metabolism, and will be increasingly produced in patients with deficiency of *p*-hydroxyphenylpyruvate oxidase. It was demonstrated to decrease considerably ascorbic acid concentration in mouse organ and blood [31]. It can impair the function of kidney tubules. Such different concentration likely suggested the more serious kidney injury happened in HUPM patients.

Quinolinic acid is an important metabolite which lies upstream of NAD+ and NADP+. Increased quinolinic acid in the kidneys of HUPM patients provided the precursor of NADPH for the biosysnthesis of polyols, dicarboxylic acids, and pyrimidine metabolism. Quinolinic acid could reduce antioxidant defenses in rat brain [32,33]. The increased quinolinic acid in HUPM patients possibly reduced the effects of antioxidants. One recent research suggested a link between quinolinic acid and kidney disease with increased proliferation when kidney mesangial cells were exposed to this compound in vitro [34]. Kim *et al *[8] performed a urine metabolomics analysis to find the correlation between kidney cancer and increased quinolinic acid. Based on previous knowledge and our results, we presumed that oxidative stress was one of the vital features in the developing of MN.

## Conclusions

Parallel metabolomics of urine and serum based on GC/MS platform is an appropriate approach for revealing the systematic alteration associated with diverse renal diseases, such as membranous nephropathy in current research. In this case, there were obvious metabolic distinctions between the patients with urine protein lower than 3.5 g/24 h (LUPM) and those higher than 3.5 g/24 h (HUPM) according to PLS-DA model analysis. In total, 26 urine metabolites and 9 serum metabolites were identified to account for such differences. The majority of differential metabolites was significantly increased in HUPM patients for both urines and serums. Combining the differential urine with serum metabolites, all metabolites were classified to 5 classes. The markedly increased metabolites (4 amino acids and citric acid) only in the serum samples of HUPM patients implied more severe injury of renal filtration function than LUPM patients. The significantly increased excretion of dicarboxylic acids, threonic acid, quinolinic acid, cholesterol, and phenolic acids in urines of HUPM patients suggested more severe oxidative stresses than LUPM patients. These results prompted us that the severity of membranous nephropathy was associated with oxidative stress. In summary, the parallel metabolomics of urine and serum is a promising approach revealing systematic metabolic variations associated with renal diseases. The urinary marker metabolites correlated with oxidative stress also showed a prospective application in early diagnosis of renal diseases.

## Methods

### Experimental participants

The experimental design involved urine samples from 14 MN patients with urine protein lower than 3.5 g/24 h (Low Urine Protein MN group, LUPM), and 15 MN patients with urine protein higher than 3.5 g/24 h (High Urine Protein MN group, HUPM). The other 3 MN patients with serum creatinine higher than 110 μmol/l but urine protein lower than 3.5 g/24 h generally have the similar clinical manifest and were classified to the HUPM group, thus 18 HUPM subjects were involved at the initial PCA analysis. All MN subjects were recruited from the outpatients of the Longhua Hospital affiliated to Shanghai University of Traditional Chinese Medicine (Shanghai, China). They were diagnosed definitely and graded by clinical manifestations, urinary protein excretion levels, serum creatinine, and percutaneous renal biopsy. After collecting, urine samples were immediately frozen, and serum samples were separated from vein blood within 1 h after blood draw. All urine and serum samples were stored at -80°C prior to sample preparation and GC/MS analysis. This experiment was approved by Research Ethics Committee of Longhua Hospital, and written informed consent was obtained from all participants.

### Chemicals

N, O-bis(Trimethylsilyl)trifluoroacetamide (BSTFA) with 1% trimethylchlorosilane (TMCS) and methoxylamine were the products of Supelco (Bellefonte, PA). High-performance liquid chromatography grade ethanol and heptane was purchased from CNW Technologies GmbH (Düsseldorf, Germany). All standard compounds, urease (Type III, from *Canavalia ensiformis*), anhydrous pyridine, and alkane standard mixture (C10-C40) were commercially obtained from Sigma-Aldrich.

### Sample preparation and derivatization

Urine samples were thawed in ice-water and short-time vortexed at room temperature. Fifty microliters of urine was incubated with 20 μl of 2000 U/ml urease at 37°C for 60 min. Then, 420 μl of ice-cold ethanol was added into the reaction solution. The mixture was vortexed for 30 s, placed at 4°C for 20 min, and subsequently centrifuged at 20 000 g at 4°C for 10 min. A quality control (QC) sample was prepared by pooling the urine samples in this study. A 392 μl of supernatant in glass vial was dried under gentle nitrogen stream, and subsequently, 50 μl of 15 mg/ml methoxylamine in pyridine was added. The resultant mixture was vortex-mixed vigorously for 30s and incubated at 37°C for 90 min to inhibit the cyclization of reducing sugars and the decarboxylation of α-keto acids. Fifty microliters of BSTFA (with 1% TMCS) were added into the mixture and derivatized at 70°C for 60 min, and subsequently a 20 μl of 200 μg/ml nonadecanoic acid methylester dissolved in heptane was added into the cooled derivatives. The final mixture was vortexed for 30s and placed in sample plate for 30 min prior to injection. At the same time, a control derivatization sample (urine was replaced by deionized water) was prepared in order to remove the background noise produced during sample preparation and GC/MS analysis.

Serum samples were thawed in ice-water and short-time vortexed at room temperature. Twenty microliters of serum was added into 60 μl of methanol-chloroform (3:1). The mixture was vortexed for 30 s, and placed in ice-water for 15 min prior to centrifugation at 20 000 g at 4°C for 10 min. A 64 μl of supernatant was transferred to a glass vial and dried under gentle nitrogen stream. The following steps are the same as the protocol of urine sample preparation.

### GC/MS analysis

The derivatized urine samples were analyzed by an Agilent 7890A gas chromatography system coupled to an Agilent 5975C MSD system with Triple-Axis Detector (Agilent, CA) as described in published literature except that the solvent delay time was set to 6.5 min [35].

### Data extraction and preprocessing

The extraction, alignment, deconvolution, and further processing of raw GC/MS data were principally referred to the previous published protocol [35]. The different parameters were time window 390-2940 s and mass window 70-600 m/z.

### Multivariate statistical analysis

The peak table (named matrix X) obtained from the section "Data extraction and Preprocessing" was imported to a commercial software Simca-P 11.0 (Umetrics, Umeå, Sweden), where multivariate statistical analysis, such as PCA and PLS-DA, was performed. All data were mean-centered and unit variance (UV)-scaled prior to multivariate statistical analysis. The PCA was used to evaluate the quality of sample analysis and holistically observe the general clustering and trends among all samples, while the PLS-DA model was used to amplify variations with a supervised means and identify the differential metabolites between two groups. The Hotelling's T2 region, shown as an ellipse in score plots of the models, defines the 95% confidence interval of the modeled variation. The quality of the models is described by the R^2^X or R^2^Y and Q^2 ^values. R^2^X or R^2^Y is defined as the proportion of variance in the data explained by the models and indicates goodness of fit. Q^2 ^is defined as the proportion of variance in the data predictable by the model and indicates predictability, calculated by cross-validation procedure [36]. A default 7-round cross-validation in Simca-P was performed throughout to determine the optimal number of principal components and avoid model over-fitting. The values of R^2^X, R^2^Y, and Q^2 ^were used as indicatives to assess the robustness of a pattern recognition model.

The differential metabolites were determined based on the combination of a statistically significant threshold of Variable Influence on Projection (VIP) values obtained from PLS-DA model and FDR adjusted *p *values from two-tailed Student's *t *test on the normalized peak areas, where the metabolites with VIP values larger than 1.0 and *p *values less than 0.05 were selected, respectively. Fold change was calculated as binary logarithm of average normalized peak area ratio between the two groups, where the positive value means that the average mass response of HUPM group is higher than LUPM group.

### Identification of metabolites

The deconvoluted spectra by our data processing protocol were introduced to the NIST MS Search 2.0 software for automatically searching compound information from the NIST 08 library and the author-constructed standard library. The searching results with match similarity larger than 80% will be accepted as candidate compounds. To obtain reliable results, a raw GC/MS data file of QC sample was imported to the AMDIS software http://chemdata.nist.gov/mass-spc/amdis/ for automatically searching against an author-constructed standard library including retention time and mass spectra. The results obtained from the above two methods were confirmed as reliable candidate compounds. The retention times of reference standards were utilized to confirm the candidate compounds. The Kovats retention indexes of remaining candidate compounds were calculated and compared with a NIST RI database and available internet database such as NIST Chemistry WebBook http://webbook.nist.gov/chemistry/.

## Competing interests

The authors declare that they have no competing interests.

## Authors' contributions

XG designed the experiments, performed the metabolomics experiments and data analysis, interpreted the results, drafted the manuscript and coordinated the study. WC designed the experiments, collected clinical samples, classified samples, and coordinated the study. RL and MW participated in sample collection and pretreatment. CC performed sample preparation and metabolomics experiments. RZ designed the experiments, interpreted the results, drafted the manuscript, and coordinated the study. YD designed the experiments, interpreted the results, and coordinated the study. All authors have read, modified, and approved the final manuscript.
